# Analysis of *pfhrp2* genetic diversity in Senegal and implications for use of rapid diagnostic tests

**DOI:** 10.1186/1475-2875-13-34

**Published:** 2014-01-29

**Authors:** Awa B Deme, Daniel J Park, Amy K Bei, Ousmane Sarr, Aida Sadikh Badiane, Papa El Hadji Omar Gueye, Ambroise Ahouidi, Omar Ndir, Souleymane Mboup, Dyann F Wirth, Daouda Ndiaye, Sarah K Volkman

**Affiliations:** 1 Laboratory of Bacteriology-Virologie, Hopital Aristide Le Dantec, Dakar, BP 7325, Senegal; 2 Broad Institute: The Broad Institute of MIT and Harvard, Cambridge, MA 02142, USA; 3 Department of Immunology and Infectious Diseases, Harvard School of Public Health, Boston, MA 02115, USA; 4 Laboratoire of Parasitology and Mycology, Faculty of Medicine and Pharmacy, Cheikh Anta Diop University, BP 5005 Dakar, Senegal; 5 School of Nursing and Health Sciences, Simmons College, Boston, MA 02115, USA

**Keywords:** *Plasmodium falciparum*, HRP2, Diversity, RDT, Senegal

## Abstract

**Background:**

The Senegalese National Malaria Control Programme has recommended use of rapid diagnostic tests (RDTs) that target the histidine-rich protein 2 (HRP2), specific to *Plasmodium falciparum,* to diagnose malaria cases. The target antigen has been shown to be polymorphic, which may explain the variability in HRP2-based RDT results reported in field studies. The genetic diversity of the *pfhrp2* gene has not been investigated in depth in many African countries. The goal of this study is to determine the extent of polymorphism in *pfhrp2* among Senegal, Mali and Uganda parasite populations, and discuss the implications of these findings on the utility of RDTs that are based on HRP2 detection.

**Methods:**

Sequencing data from the *pfhrp2* locus were used to analyze the genetic diversity of this gene among three populations, with different transmission dynamics and malaria parasite ecologies. Nucleotide diversity (π) and non-synonymous nucleotide diversity (π_NS_) were studied in the *pfhrp2* gene from isolates obtained in Senegal. Amino acid repeat length polymorphisms in the PfHRP2 antigen were characterized and parameters of genetic diversity, such as frequency and correlation between repeats in these populations, were assessed.

**Results:**

The diversity survey of the *pfhrp2* gene identified 29 SNPs as well as insertion and deletion polymorphisms within a 918 bp region. The Senegal *pfhrp2* exhibited a substantial level of diversity [π = 0.00559 and π_NS_ = 0.014111 (π_S_ = 0.0291627)], similar to several polymorphic genes, such as *msp1*, involved in immune responses, and the gene encoding the SURFIN polymorphic antigen, which are surface exposed parasite proteins. Extensive repeat length polymorphisms in PfHRP2, as well as similar patterns in the number, organization and the type of predicted amino acid repeats were observed among the three populations, characterized by an occurrence of Type 2, Type 4 and Type 7 repeats.

**Conclusions:**

These results warrant deeper monitoring of the RDT target antigen diversity and emphasize that development of other essential genes as a target for diagnostic tools is critical.

## Background

For over 50 years various strategies have been developed to reduce the burden of *Plasmodium falciparum*, the most virulent malaria species and the primary cause of malaria-related mortality across the globe [1]. International funding programmes have first tasked the eradication of this disease and, as a follow-up for regional elimination, have provided tools such as insecticide-treated bed nets (ITNs), indoor residual spraying (IRS), artemisinin-based combination therapy (ACT), and rapid diagnostic tests (RDTs) to many malaria-endemic regions. This report focuses on the implications of genetic diversity in the *pfhrp2* gene that may compromise the use of RDTs for the diagnosis of malaria.

In 2006, the Senegalese National Malaria Control Programme (NMCP) scaled up the use of malaria RDTs at all health facilities in Senegal for case management [2] with the ultimate goal being to treat all (and only) true malaria infections, as misdiagnosis of malaria can contribute to drug resistance [3]. The number of RDTs currently used in Senegal is between 900,000 and 1,200,000 annually [4]. RDTs are ideal for rural conditions in developing countries where well-equipped health facilities and expert microscopists are lacking, as they give rapid results, require no electrical equipment or temperature-sensitive reagents, and can be performed without extensive training. The technical performance of these tests has been described previously [5], and although they are sensitive and specific to detect most cases of malaria at parasitaemia greater than 200 parasites/μl, product performance can vary widely at low parasitaemia [6], and these tools may miss individuals harbouring a very low parasitaemia.

RDTs differ in the antigens they detect. Some are based on detection of the *P. falciparum* specific histidine-rich protein-2 (PfHRP2), or *P. falciparum* specific lactate dehydrogenase (PfLDH), while others recognize antigens common to *P. falciparum*, *Plasmodium vivax, Plasmodium ovale,* and *Plasmodium malariae* (pan-species pLDH and aldolase) [7]. *Plasmodium* aldolase and lactate dehydrogenase (pLDH) are highly conserved RDT targets, [8,9] in contrast to the *P. falciparum* PfHRP2 which is considered to be more variable [10,11]. In Senegal, HRP2-based RDTs are most commonly used in health facilities because of their previously demonstrated sensitivity and specificity and because *P. falciparum* represents the major *Plasmodium* species (more than 98%) in Senegal [12], with *P. malariae* and *P. ovale* comprising less than 2% of malaria cases*,* and no *P. vivax* (0%) observed in 2013 [4].

The *pfhrp2* gene of the *P. falciparum* 3D7 is localized on chromosome 8 (PF3D7_0831800, PlasmoDB Version 9.3) and contains two exons separated by an intron. The first exon encodes a signal peptide and the second exon contains a protein-export motif followed by specific histidine-rich repeats, which have been described previously [10,11]. PfHRP2 is synthesized throughout the asexual life cycle and in early sexual stages of *P. falciparum*[13]. The protein is characterized by the presence of a number of variable tandem repeats, each one named with a Type based on the motif being repeated [10,11].

Specific amino acid repeats AHHAAD (Type 7), AHHAHHAD (Type 2) have been described as possible epitopes targeted by the monoclonal antibodies used to detect HRP2 in some RDTs [14]. Many studies of the genetic diversity of this antigen show extensive variation within isolates of the same country and between isolates of different countries [10,11,15]. In Senegal, the genetic diversity of the gene target by the RDT has not yet been investigated in depth. The goal of this study is to determine the extent of genetic polymorphism in the *pfhrp2* gene in a Senegalese population in comparison with two other diverse African populations: Mali and Uganda. Indeed, previous reports from Mali have indicated the loss of the HRP2 locus from some parasites, and this study investigates whether changes in this locus among Senegalese parasites may compromise the performance of the RDTs so widely used for malaria diagnosis.

To accomplish this goal, the *pfhrp2* gene was sequenced using patient samples from Senegal, and along with available sequencing data from Mali and Uganda (three endemic African countries with different malaria ecologies and epidemiology), was used to assess the nucleotide polymorphism as well as the distribution of specific PfHRP2 amino acid repeats.

## Methods

### Parasite isolates

Both the Ethics Committee of the Ministry of Health in Senegal and the Institutional Review Board of the Harvard School of Public Health approved this study. Parasite isolates from Senegal used in this study were collected from malaria febrile patients, after informed consent, during the malaria transmission season (August-December), from 2001 to 2010, at three health facilities: Pikine, Thies and Velingara. Pikine, a suburban area (approximately 15 km from Dakar) is classified as low transmission area (<5 cases per 1,000 individuals). Thies (70 km from Dakar), is a hypo-endemic urban site with an average of five malaria cases per 1,000 people. For Pikine and Thies, the entomological inoculation rate (EIR) is low less than five infectious bites per year. Velingara (in the south of Senegal, 565 km from Dakar) is a highly endemic area, with the level of transmission approximately 21 malaria cases per 1,000 people and the EIR around 100 to 300 infectious bites per year [16]. Overall, 54 Senegal isolates were used in this study (ten isolates from Pikine, 40 isolates from Thies and four isolates from Velingara).

Mali is a border country of Senegal. The samples used in this study are from Bandiagara in central eastern Mali, where malaria is endemic and the *P. falciparum* transmission is intense with seasonal peaks in July-October [1]. Uganda is located in East Africa and is highly endemic for malaria, which is the leading cause of morbidity and mortality [17]. However, samples were collected in Kampala, an urban centre in Uganda where malaria is meso-endemic, arising perennially with peaks during the two rainy seasons from August to December and from February to June [17].

### DNA extraction

*Plasmodium falciparum* isolates from Senegal were culture-adapted and deposited at the MR4 reagent repository. Genomic DNA was isolated, for sequencing, using Qiagen reagents according to the manufacturer instructions.

### DNA genotyping, sequencing and DNA translation

Samples were determined to be monogenomic and genetically distinct by a 24 single nucleotide polymorphism (SNP) molecular barcode [18]. Genomic DNA was sequenced using Illumina Hi-Seq machines, aligned to the 3D7 reference assembly, and variants were identified using previously described methods [19]. Using variant and invariant sites, the *pfhrp2* gene coding sequence (spliced CDS sequence) was reconstructed for each sample, inserting an ‘N’ to represent an undetermined base for sites that could not be resolved due to insufficient sequence coverage. The SNP calls for the Uganda and Mali isolates are downloadable from the Broad website [20].

Two isolates from Senegal SenT113.09 and SenV042.05 were PCR re-sequenced across the *pfhrp2* region to confirm a frameshift mutation identified with GATK software analysis of the sequence data (Additional file 1). Primers specific to *pfhrp2* amplifying the frameshift position (nucleotides position 571 and 574 of SenT113.09 and SenV042.05 respectively) were designed (Forward: **GCAC**GCCGTTTTTGCCTCCGTACT and Reverse: **GCAC**GCAATGTGTGGCGGCTTC). PCR amplification was carried out in a final reaction volume of 30 μl containing DNA template (100 ng), primers (50 μM for each primer), dNTPs (10 mM for each dNTP), and 0.6 μl Polymerase (Pfu Ultra II Fusion polymerase, Agilent Technologies, Inc). PCR amplification conditions were: denaturation at 95°C for 2 min, followed by 35 cycles of 95°C for 25 sec, 55°C for 25 sec and 60°C for 90 sec, with a final extension phase at 72°C for 10 min. Quality control of PCR reactions was performed by gel electrophoresis. PCR products were purified with Qiagen columns and sequenced in both the forward and reverse directions using the same PCR primers as were used for the amplification. DNA sequencing was performed by GENEWIZ [21] and amino-acid sequences were deduced from the nucleotide sequences obtained using TRANSEQ [22]. Nucleotide and amino acid sequences were aligned with the 3D7 reference sequence (PlasmoDB MAL7P1.231) using Bio Edit Sequence Alignment Editor Software (version 7.1.3.0). Polymorphic repeats were characterized as described [15].

### Nucleotide diversity

The genetic diversity of the *pfhrp2* gene was measured for the Senegal population (n = 54) using the nucleotide diversity (π), which quantifies the average number of nucleotide differences per site between DNA sequences among the sample population; and the π_NS_ and π_S_ (non-synonymous and synonymous polymorphism, respectively) values were calculated by dividing the average number of non-synonymous or synonymous pairwise differences by the number of non-synonymous or synonymous sites computed for all genes in the *P. falciparum* genome (Additional file 2) [23]. For Mali (n = 11) and Uganda (n = 9) isolates, DnaSp [24] was used to predict nucleotide diversity (π) due to a smaller than ideal sample size for estimating SNP diversity.

### Statistical analysis

Differences in the median number of each type of amino acid repeat were tested by Kruskall-Wallis for Senegal, Mali and Uganda *pfhrp2* sequences for each individual repeat (α = 0.05). The Spearman rank test was used to evaluate the correlation between pairs of repeats in a given population. For all statistical tests a significance threshold of α = 0.05 was used.

## Results

### Analysis of the nucleotide diversity in the *pfhrp2* gene

**Figure 1:**
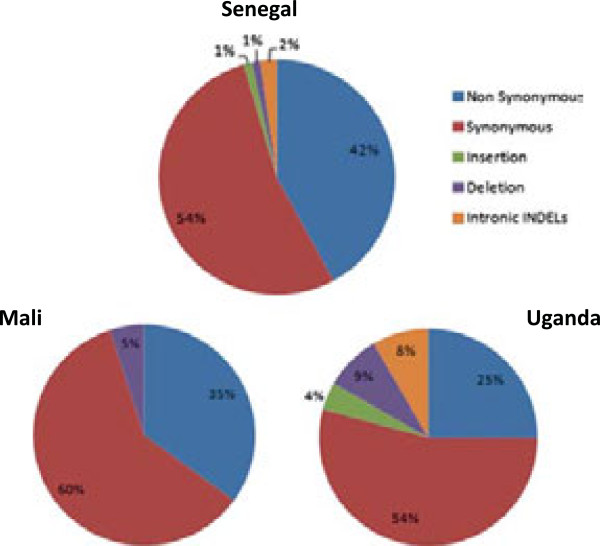
**Frequency of polymorphism occurrence in the*pfhrp2*gene from Senegal, Mali, and Uganda isolates.** Non-synonymous, synonymous, coding indels polymorphisms were observed. Synonymous and non-synonymous SNPs are the more common polymorphism in the three populations. Synonymous SNPs are higher than non-synonymous SNPs, showing variability between countries. Intronic indels and coding insertion were not observed in Mali.

A total of 67 polymorphic sites among the 74 isolates were identified, with 29 of these polymorphisms described as “singletons”, appearing only in one isolate, ten as “doubletons” found in two samples, and 28 as “multiples” that appear in at least in three or more samples (Additional file 1). The full-length CDS of the *pfhrp2* gene ranges from 900 to 921 bp, and sequences were aligned using Clustal W (Additional file 3). Polymorphisms in the *pfhrp2* sequence from the three countries include: 35 synonymous and 20 non-synonymous SNP positions, as well as 11 insertion-deletions (indels) (Figure 1). For all defined populations, synonymous SNPs are more common than non-synonymous SNPs (Figure 1), and polymorphism varies within population, showing an intra- and inter-variability between isolates.

**Figure 2:**
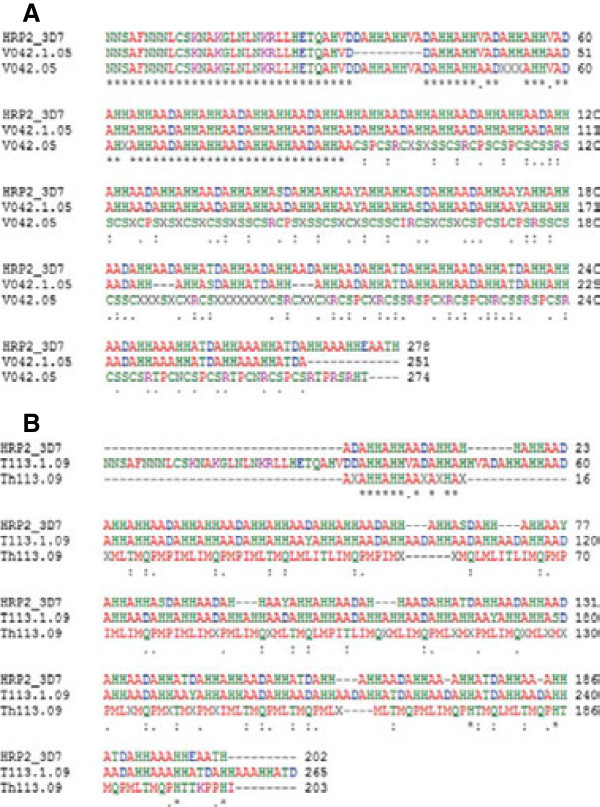
**PCR re-sequencing.** To confirm the frameshifting polymorphisms in isolates SenTh113.09 and SenV042.05, PCR amplification and re-sequencing of the region surrounding the polymorphic positions corresponding to aa571 and aa574 in the coding sequence were performed. **A**: Alignment of the re-sequenced SenV042.1.05 PfHRP2 sequences with 3D7 isolate. The two sequences share 91.1% identity. **B**: Alignment of the re-sequenced SenTh113.1.09 PfHRP2 sequence with 3D7 isolate showed 79.7% identity. These results suggest that errors in Illumina sequencing or GATK’s Unified Genotyper produced a false call in these two cases.

Of the 11 indels called by sequencing data, seven are predicted to result in insertions or deletions of entire codons (i.e. a multiple of three bp), two are intronic and two are suggestive of frameshifts within the coding sequence. The two frameshifting indels are both singletons and existed only in isolates SenT113.09 (insertion of two nucleotides) and SenV042.05 (deletion of two nucleotides). To confirm these frameshifts, the two isolates were PCR re-sequenced (with the new sequences being named SenT113.1.09 and SenV042.1.05). The amplification of genomic DNA by primers surrounding polymorphic amino acid (aa) positions 571 and 574, gave an amplicon of approximately 900 bp in length. The alignment of the translated PfHRP2 protein (Figure 2) revealed an identity between the new sequences SenT113.1.09 and SenV042.1.05 (79.7 and 91.1%, respectively) with the 3D7 reference, and the lack of any frameshift, suggesting that errors in Illumina sequencing or GATK’s Unified Genotyper produced a false call in these two cases. Consequently, the effects of the two frameshifting indels were ignored in further analysis.

**Figure 3:**
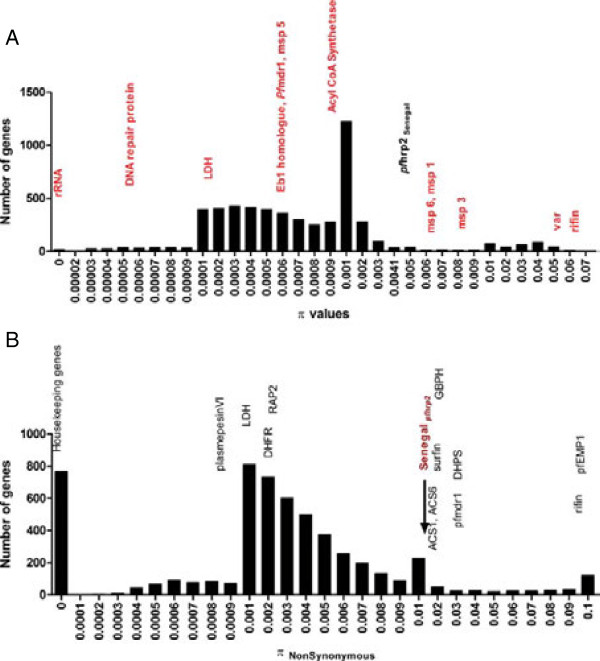
**Nucleotide diversity (π) and non-synonymous polymorphism (π**_**NS**_**). A**: Comparison of the nucleotide (π) of *pfhrp2* in the Senegalese population, to other *P. falciparum* genes. All π values were rank in the *P. falciparum* genome π = 0.0000 to π = 0.07. The diversity π *pfhrp2* = *0.00559* for Senegal falls within regions containing polymorphic genes such as *msp1* or *msp6.***B**: The non-synonymous polymorphism (π_NS_ = 0.014111) for *pfhrp2* gene showed that 89% of genes have a lower π_NS_ than *pfhrp2*.

The nucleotide diversity (π) and π_NS_ diversity of the *pfhrp2* gene were then studied in depth among Senegalese isolates to investigate whether *pfhrp2* was remarkable in this population, since at a protein level a high π_NS_ may affect the PfHRP2 protein detection. Thus, *pfhrp2* π and π_NS_ were compared to other genes in the *P. falciparum* genome. As a point of reference, housekeeping genes such as 18S RNA (PF3D7_1371000) have zero nucleotide diversity π = 0.000 and π_NS_ = 0.000 (Figure 3). The nucleotide diversity for *pfhrp2* is π = 0.00559, falling near highly polymorphic genes such as merozoite surface proteins (*msp6, msp1,* π *>* 0.007) (Figure 3A). The distribution of π_NS_ varies widely in frequency among *P. falciparum* genes (Figure 3B, Additional file 2) and 89% of *P. falciparum* genes have a lower π_NS_ than *pfhrp2*. The π_NS_ = 0.0141 (π_S_ = 0.0291627) for *pfhrp2* is similar to several genes encoding SURFIN antigens (PF3D7_0800700) involved in cyto-adherence (π_NS_ = 0.014065) and a *glycophorin binding protein* family (PF3D7_1401000) (π_NS_ = 0.01574).

For Mali (n = 11) and Uganda (n = 9) isolates, the predicted nucleotide diversity for *pfhrp2* using DnaSp, revealed π = 5.76×10^-3^ and π = 1.065×10^-2^ for Mali and Uganda, respectively, which were both greater than Senegal *pfhrp2* (π = 0.00559).

### Analysis of the PfHRP2 amino acid repeat polymorphisms

The length of the PfHRP2 for the reference strain 3D7 is 305 amino acids. Amino acid sequences were deduced from the nucleotide sequences and aligned using BioEdit (Clustal W). Variations in the length of the PfHRP2 sequences were observed. These fragments ranged in size from 299–307 aa (Additional file 4), with an average length of 303 aas.

**Figure 4:**
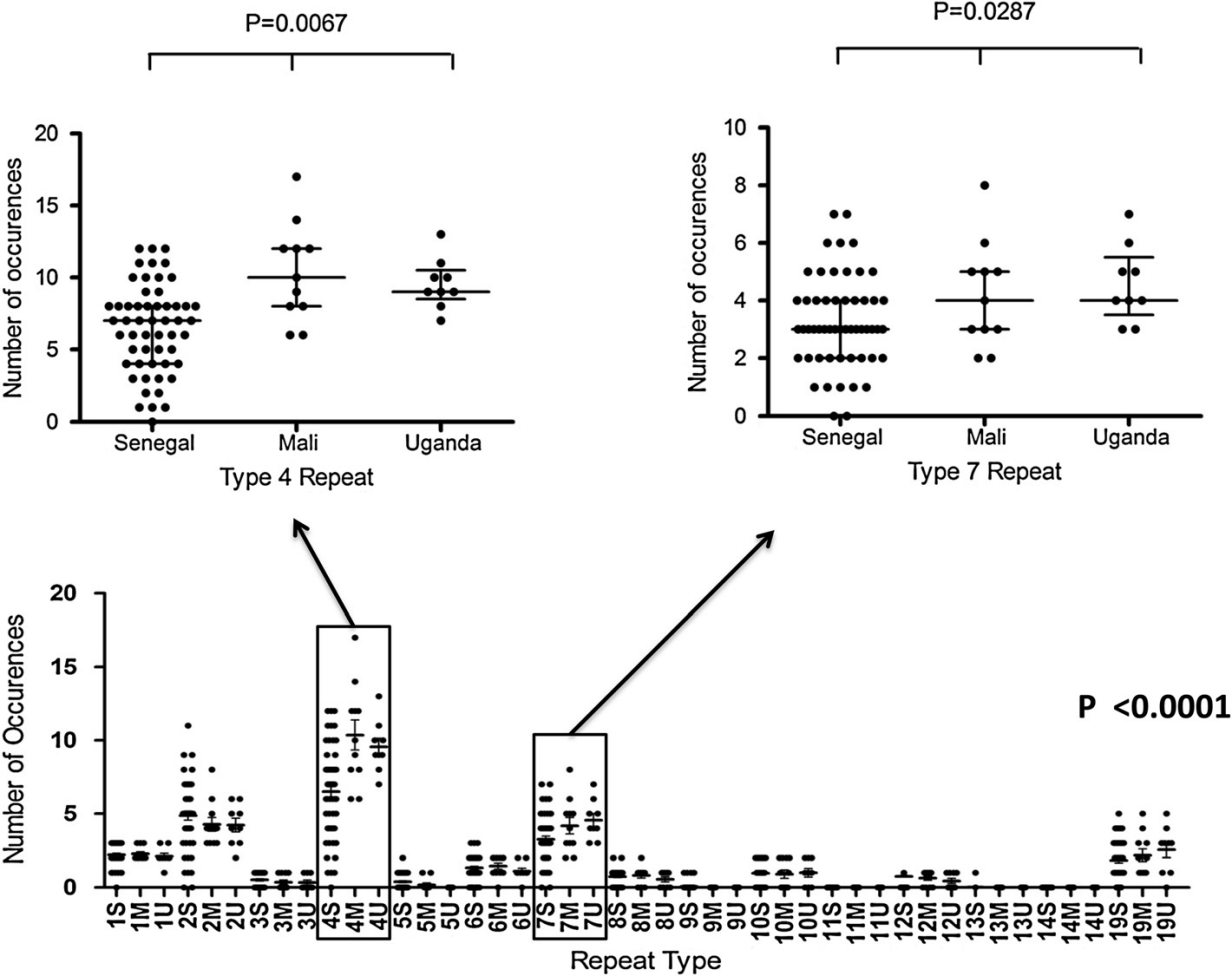
**Variation in the number of types repeats.** Differences in the median number of repeats of each type are shown. There is a significant difference in the median of the Type 4 repeat (P value = 0.0067) and the Type 7 repeat (P value = 0.0287) within the three African populations (S: Senegal, M: Mali, U: Uganda) of *P. falciparum*; P value <0.0001). Population comparison using Kruskall Wallis in a non-parametric way shows a significant difference in the median number of the Type 4 and the Type 7 repeats. The frequency and distribution of amino acids repeats shows inter and intra-species variation in the PfHRP2 sequence.

The PfHRP2 protein is composed of a varying number of amino acids repeats [15]. The motif VLSAAVFASVLLLDNNNSAFNNNL (termed Type 0), is conserved in all Senegalese, Malian and Ugandan PfHRP2 sequences (Table 1). In this study, 13 of the 20 different amino acid repeats previously reported were detected (Table 1) [10,15]. PfHRP2 sequences begin with the Type 1 (AHHAHHVAD) motif and end with the Type 12 (AHHAAAHHEAATH) motif (Additional file 4). The frequency distributions of PfHRP2 type repeats within the three African populations show an overall significant difference in the median number of occurrences of the different repeat types for each of the three populations (Kruskall-Wallis, p < 0.0001) (Figure 4). The frequencies of Type 4 and Type 7 repeats, repeats thought to be the targets of RDTs, were significantly different between the three populations (Figure 4) (Kruskall-Wallis, p = 0.0067, p = 0.0287, respectively) suggesting that the frequency and distribution of amino acid repeats could possibly reflect population level variation in the PfHRP2 protein.

**Table 1:** The frequency of amino-acid repeats of PfHRP2

**Types**	**Repeat found in the HRP2 sequence**	**Repeat frequency in Senegal isolates N = 54**	**Repeat frequency in Mali isolates N = 11**	**Repeat frequency in Uganda isolates N = 9**
0	VLSAAVFASVLLLDNNNSAFNNNL	**54 (100%)**	**11 (100%)**	**9 (100%)**
1	AHHAHHVAD	52 (96.29%)	**11 (100%)**	**9 (100%)**
2	AHHAHHAAD	51 (94.4%)	**11 (100%)**	**9 (100%)**
3	AHHAHHAAY	29 (53.7%)	5 (45.45%)	7 (78%)
4	AHH	52 (96.29%)	**11 (100%)**	**9 (100%)**
5	AHHAHHASD	20 (37.07%)	2 (18.18%)	0%
6	AHHATD	45 (83.33%)	10 (90.9%)	**9 (100%)**
7	AHHAAD	51 (94.44%)	**11 (100%)**	**9 (100%)**
8	AHHAAY	37 (68.51%)	8 (72.72%)	**9 (100%)**
9	AAY	3 (5.55%)	0%	0%
10	AHHAAAHHATD	33 (61.1%)	6 (54.54%)	**9 (100%)**
11	AHN	0%	0%	0%
12	AHHAAAHHEAATH	40 (74.07%)	7 (63.63%)	**9 (100%)**
13	AHHASD	1 (1.85%)	0%	1 (11%)
14	AHHAHHATD	0%	0%	0%
19	AHHAA	43 (79.62%)	**11 (100%)**	8 (88.88%)
20	SHHD	0%	0%	0%
21	AHHAHHATY	0%	0%	0%
22	AHHAHHAGD	0%	0%	0%
23	ARHAAD	0%	0%	0%
24	AHHTHHAAD	0%	0%	0%

Repeat Types found in Senegal, Mali, and Uganda isolates. Repeat Types found in 100% of isolates are in bold, 0% and other percentages are in plain text.

The overall frequency of the Type 2, Type 4 and Type 7 repeats were compared to other regions of PfHRP2. The Type 2 (range 0–11), Type 4 (range 0–17) and Type 7 (range 0–8) were among the more variable repeats that also had the highest frequencies in this study for Senegal, Mali and Uganda (Figure 3 and Additional file 5). The other repeat types showed lower frequencies (range 0–3). Similar results were reported in other studies [11,25] where the Type 2 (range 2–14) and Type 7 (range 0–4) were the most frequent types observed compared to other repeat types [25].

Senegal isolates lack Type 11 and Type 14 repeats, as do Mali and Uganda isolates. However, 5.5% of Senegal isolates have the Type 9 repeat, which is absent from Mali and Uganda samples. The Type 13 was not observed in Mali isolates and one isolate from both Senegal and Uganda contained this motif (Table 1). Similarly, while the Type 5 repeat is absent in Uganda isolates, it is present in 37.07% of Senegal samples and in 18.18% of Mali samples (Table 1, Figure 3).

In addition to the frequency of the repeats, a difference in the organization of the repeats in the PfHRP2 sequences was observed. Some Types are always found in the same position in the protein: Type 3 (AHHAHHAAY) at aa168-176; Type 5 (AHHAHHASD) at aa177-185; Type 8 (AHHAAY) at aa192-197; Type 9 (AAY) at aa192-197; and, Type 12 (AHHAAAHHEAATH) at aa289-301. Others motifs such as Type 1 (AHHAHHVAD), Type 2 (AHHAHHAAD), Type 4 (AHH) and Type 10 (AHHAAAHHATD) can be present in clusters of two, three or five copies and are distributed throughout the protein (Additional file 4). When present, Type 1 and Type 10 motifs are found with at least two copies that are always repeated in tandem, at aa57-83 and aa267-aa288, respectively. Interestingly, the extent of Type 2 tandem repeats varies between isolates, and can be observed as a cluster of tandem repeats, or smaller clusters separated by other repeats. Type 4 is mostly present in two tandem repeats usually at position aa75-80, but is also found at other positions in the protein. The Type 6 repeat (AHHATD) most often occurs in three positions (aa213-218; aa237-242; aa252-257) while Type 7 (AHHAAD) and Type 19 (AHHAA) are widely dispersed in PfHRP2.

In addition to variation between populations in repeat structure and frequency, there was a significant correlation between repeat Type 1-Type 2 and Type 3-Type 5 (rs = 0.6158 P < 0.0001, rs = 0.5151 P < 0.0001, respectively; Bonferroni correction P <1.84 10^-4^) in the Senegalese PfHRP2 sequences. For Mali PfHRP2 Types repeats, a significant correlation was observed between repeat Type 10-Type 19 (rs = −0.8945, P < 0.0001). No significant correlation was observed in repeat types from among Ugandan sequences, possibly due to the small sample size in Uganda (n = 9) which makes statistical interpretation more challenging (Table 2).

**Table 2:** Correlations found between repeat types

**Country**	**Type repeats correlated**	**rs**	**P value**
Senegal	Type 1 vs Type 2	0.6158	< 0.0001
	Type 3 vs Type 5	0.5151	< 0.0001
Mali	Type 10 vs Type 19	− 0.8945	< 0.0001
Uganda	No statistical correlation found		

Spearman rank analysis was performed on all pairs of repeat motifs in a given population. The Bonferroni correction was applied. The total number of tests is n = 273 for the three populations and for all statistical tests α = 0.05, given a threshold P <1.83 × 10^-4^. Significant correlations are listed. (vs: *versus*, rs: Spearman rank).

## Discussion

*Plasmodium falciparum* tests targeting the PfHRP2 antigen have demonstrated high detection rates [26], however test performance showed variability between lots and between similar products. Recently *P. falciparum* genetic factors have been investigated as a possible cause of variability in RDT detection rates [15,25,27]. As genetic diversity in *P. falciparum* is associated with transmission intensity, such findings could significantly interfere with the ability to accurately detect all malaria cases, and therefore have great potential to impact control strategies and efforts to eliminate or eradicate the disease [28]. For this reason, it has become increasingly important to characterize the diversity of target antigens used in RDTs to predict the impact genetic variation has on diagnosis, since it has the potential to affect the sensitivity of these diagnostic tests. In this study, the genetic diversity (π and π_NS_) in the *pfhrp2* gene was described and polymorphisms in the PfHRP2 protein among parasites obtained from human infections in three African countries with distinct patterns of malaria transmission were analyzed, to provide additional information about the diversity of this locus to that described previously [10,11,25].

Here, the global nucleotide diversity π values calculated for *pfhrp2* in the Senegal, Mali and Uganda isolates are similar to highly polymorphic genes families exposed to strong immune pressure, such as *msp1* and *msp6*, since these genes are highly polymorphic [29]. In addition, the π_NS_ for the *pfhrp2* is high, similar to π_NS_ for genes encoding the polymorphic SURFIN antigens expressed on *P. falciparum* merozoite and infected erythrocytes [30]. The *pfhrp2* gene is located in the subtelomeric region of chromosome 8 within a dynamic area where recombination events frequently contribute to the high genetic variation observed among genes from these regions [31]. In addition, the *pfhrp2* gene reveals differential polymorphism by geography, with distinct population profiles of synonymous and non-synonymous SNP variants, with over half these variants being synonymous SNPs. This is particularly important because non-synonymous SNP may destabilize protein structure or interfere with either ligand binding or the formation of domain-domain interfaces [32] and these changes may affect the ability of monoclonal antibodies to detect PfHRP2, thus compromising the sensitivity of the RDT.

Insertions and deletions of nucleotides observed in this study provided similar level of polymorphisms as SNPs in *pfhrp2*. Previous studies have reported deletion of the *pfhrp2* gene associated with false-negative HRP2-based RDTs in Mali, suggesting that spontaneous *hrp2* deletions could be one mechanism of genetic variation responsible for RDT failures [27]. Such deletions have been reported before in South America, where 15/275 parasites (5.5%) were found with deletions of the *pfhrp2* gene, generating concerns over the reliability of PfHRP2-based RDTs in these regions [33]. Furthermore, genetic deletions were not restricted to the *pfhrp2* alone, but were found to extend into neighbouring genes such as Hsp70 (PF3D7F_0831700) in the 3’ region of *pfhrp2* and in 5’ a pseudo-gene encoding a *Plasmodium* exported protein (PF3D7F_0831900) [34]. Such data emphasize the importance of precisely mapping deletions in *pfhrp2* in parasites from Senegal and other geographic regions.

It is important to note that the genomic DNA samples used in this study were obtained from culture-adapted *P. falciparum* isolates (and additionally from single clone infections). Further studies should focus on DNA obtained directly from patients as many infections are polygenomic, and also culturing parasites can lead to artifactual differences resulting from the process of culture adaptation, such as breakage of *P. falciparum* chromosomes occurring frequently in the *pfhrp2*, a subtelomeric gene [35]. However, the number of isolates was quite small for Mali and Uganda to allow more stringent comparison. Nevertheless, these findings suggest that the parasite differences based upon different geography, and consequently ecology and epidemiology, are important to consider.

At the amino acid level, the PfHRP2 protein showed significant differences, in the organization and the number of repeats, likely due to chromosome breakage and healing occurring randomly in a site-unspecific manner during culture *in vitro*[35]. The composition of the PfHRP2 sequence repeats in Senegalese isolates is similar to Mali and Uganda isolates where the Type 2 (AHHAHHAAD), Type 4 (AHH) and the Type 7 (AHHAAD) are the mostly common repeats (Table 1). The antigen PfHRP2 differs in the composition and the number of repeat types compared to parasites from global endemic countries previously reported [10,11,15]. Specifically, Senegalese PfHRP2 isolates differ from Cameroon isolates (presence of Type 11, and Type 14 repeats), Thailand (presence of Type 14 repeat), Philippines (presence of Type 11 repeat), and Papua New Guinea (presence of Type 14 repeat). In this analysis Type 11 and Type 14 repeats were not observed. Additionally, Type 20 (SHHDD), Type 21 (AHHAHHATY), Type 22 (AHHAHHAGD), Type 23 (ARHAAD), and Type 24 (AHHTHHAAD) repeats reported previously [10] were not found.

These results agree with previously published studies [10,25], which found a high frequency of Type 2 and Type 7 repeats. Ideally, for a universal and sensitive RDT, the target epitope should be present in all parasites, regardless of specific population, and should be present in multiple copies [14]. For RDT sensitivity, a major factor is the binding specificity and affinity of the monoclonal antibodies for the target epitopes, thus the expression level and number of these epitopes could potentially influence the binding affinity. From these data and that of other studies, the high frequency of Type 2, Type 4 and Type 7 repeats observed in *P. falciparum* isolates could contribute to the sensitivity of the RDTs in the African populations studied.

The study of the amino acid composition of the PfHRP2 protein showed that different factors can contribute to polymorphism at the protein level: both the organization of the repeats within sequences and the position of repeats in the antigen. In fact, the frequency or distribution of the target epitopes present in a particular parasite population may have an impact on the efficiency of antigen detection in this population and the sensitivity of RDTs [10,15]. These parameters should be taken into account in the choice and the design of monoclonal antibodies that could be used for PfHRP2 based RDTs.

## Conclusion

To counter increasing malaria drug resistance and to achieve the goal of malaria eradication, the need for reliable diagnostic tools is critical. This study provides information about the genetic diversity of the *pfhrp2* gene and the predicted polymorphism in the PfHRP2 antigen from sequences obtained from three African parasite populations Senegal, Mali and Uganda. For all three populations of parasites, the higher rate of synonymous SNPs in *pfhrp2* suggests evolutionary selection of this SNP. However, for Senegal isolates, the π and π_NS_ warrant deeper follow-up investigations as they may represent functional variation, which would potentially interfere with RDT detection. *Pfhrp2* is polymorphic at the nucleotide level, shown by both synonymous and non-synonymous SNPs; nucleotide (π) and non synonymous (π_NS_) diversity similar to highly polymorphic genes such as *msp1* and gene encoding SURFIN antigen. PfHRP2 showed extensive repeat length polymorphisms with an occurrence of the Type 2, Type 4 and Type 7 repeats. Similar patterns were observed in the number, organization and the type of amino acid repeats in the protein among the three African populations. However, the PfHRP2 showed variation within isolates of the same country and between isolates of different countries, addressing concern in the efficacy of the HRP2 based RDT test. Since the polymorphism described in the target antigen is a recognized concern, comprehensive studies of possible factors influencing the sensitivity of these diagnostic tools, including genetic deletions and protein expression level, are critical in predicting the efficacy of RDTs in global populations.

## Abbreviations

NMCP: National Malaria Control Programme; RDTs: Rapid diagnostic tests; HRP-2: Histidine-rich proteins-2; PCR: Polymerase chain reaction; WHO: World Health Organization; aa: Amino acid; EIR: Entomological inoculation rate; rs: Spearman rank; ACT: Artemisinin-based combination therapy; ITNs: Insecticide-treated bed nets; IRS: Indoor residual spraying; SNP: Single nucleotide polymorphism; CDS: Coding sequence; MSP: Merozoite surface protein.

## Competing interests

The authors declare that they have no any competing interests.

## Authors’ contributions

Data analysis, manuscript writing, and PCR re-sequencing were performed by ABD. DP provided the sequencing data for analysis and reviewed the manuscript. AKB gave constructive advice on analysis, assisted with writing and revision of the manuscript. OS, ASB, POG, and AA reviewed the manuscript. SKV performed culture-adaptation of the Senegal and Mali parasites and prepared DNA for sequencing from these parasite populations, and assisted with the writing and revision of the manuscript. DN led the Senegal field collection sites for samples collected. DP and SKV guided the analysis performed by ABD. All authors have read and approved the final version of manuscript.

## Supplementary Material

Additional file 1 **Polymorphic position in the *pfhrp2 *** **gene.** The positions of the SNPs identified are showed. The Table contains information about the SNP types, the description of the gene where the SNP is located, the position of the SNPs, the possible alternative alleles, the minor allele frequency, and the call rate. A total of 67 polymorphisms were identified, one per row, among Senegal, Mali and Uganda isolates. These polymorphisms include SNPs and indels. The indels position is reported as the position before the insertion/deletion point, and the base before it is repeated. The first letter in the isolate names indicates the site of collection (P for Pikine; V for Velingara; and, T for Thies), and the last two digits in the isolate names indicate the year of collection (e.g., P05.02 was isolated in 2002). Click here for file

Additional file 2 **Nucleotide diversity synonymous and non-synonymous polymorphism in Senegalese isolates.** The Table contains 5,495 *P. falciparum* genes from the set of Senegalese isolates. Each line corresponds to a description of the gene with data on localizations in the genome, the nucleotide diversity π, synonymous and non-synonymous polymorphism (π_S_ and π_NS_) and additionally the ratio (π_NS_/π_S_). Click here for file

Additional file 3 **Alignment of *pfhrp2 *** **coding sequences from, Senegal, Mali and Uganda isolates.** All CDS were aligned Using BioEdit software, with Clustal W program. Click here for file

Additional file 4 **Alignment of PfHRP2 sequence.** All protein sequences were aligned against the 3D7 reference, using BioEdit software and Clustal W program. Click here for file

Additional file 5 **PfHRP2 Repeat Types.** Thee table contains the repeat Types found in the PfHRP2 sequence from Senegal, Mali and Uganda isolates. Each line represents an isolate, with the number of the different repeat Types observed within the sequences indicated. Click here for file
