# Supplementary material for: Analysis of pfhrp2 genetic diversity in Senegal and implications for use of rapid diagnostic tests
Source: Malar J. 2014 Jan 29;13:34. doi: 10.1186/1475-2875-13-34 (PMC3913323; doi:10.1186/1475-2875-13-34)
Supplement: Additional file 3 — Alignment of pfhrp2 coding sequences from, Senegal, Mali and Uganda isolates. All CDS were aligned Using BioEdit software, with Clustal W program. [file 1475-2875-13-34-S3.docx]

Additional File 3 Deme
