# Supplementary material for: Analysis of pfhrp2 genetic diversity in Senegal and implications for use of rapid diagnostic tests
Source: Malar J. 2014 Jan 29;13:34. doi: 10.1186/1475-2875-13-34 (PMC3913323; doi:10.1186/1475-2875-13-34)
Supplement: Additional file 4 — Alignment of PfHRP2 sequence. All protein sequences were aligned against the 3D7 reference, using BioEdit software and Clustal W program. [file 1475-2875-13-34-S4.docx]

Additional File 4 Deme
